# Automated Segmentation and Object Classification of CT Images: Application to *In Vivo* Molecular Imaging of Avian Embryos

**DOI:** 10.1155/2013/508474

**Published:** 2013-08-12

**Authors:** Alexander Heidrich, Jana Schmidt, Johannes Zimmermann, Hans Peter Saluz

**Affiliations:** ^1^Department of Cell and Molecular Biology, Leibniz Institute for Natural Product Research and Infection Biology, Hans Knöll Institute, Beutenberg Straße 11a, 07745 Jena, Germany; ^2^Institut für Informatik/I12, Technische Universität München, Boltzmannstrraße 3, 85748 Garching bei München, Germany; ^3^Definiens AG, Bernhard-Wicki-Straße 5, 80636 München, Germany; ^4^Friedrich Schiller University of Jena, Fürstengraben 1, 07743 Jena, Germany

## Abstract

*Background*. Although chick embryogenesis has been studied extensively, there has been growing interest in the investigation of skeletogenesis. In addition to improved poultry health and minimized economic loss, a greater understanding of skeletal abnormalities can also have implications for human medicine. True *in vivo* studies require noninvasive imaging techniques such as high-resolution microCT. However, the manual analysis of acquired images is both time consuming and subjective. *Methods*. We have developed a system for automated image segmentation that entails object-based image analysis followed by the classification of the extracted image objects. For image segmentation, a rule set was developed using Definiens image analysis software. The classification engine was implemented using the WEKA machine learning tool. *Results*. Our system reduces analysis time and observer bias while maintaining high accuracy. Applying the system to the quantification of long bone growth has allowed us to present the first true *in ovo* data for bone length growth recorded in the same chick embryos. *Conclusions*. The procedures developed represent an innovative approach for the automated segmentation, classification, quantification, and visualization of microCT images. MicroCT offers the possibility of performing longitudinal studies and thereby provides unique insights into the morpho- and embryogenesis of live chick embryos.

## 1. Background

The *in ovo *chick embryo is a highly versatile model organism with a long history of use in biological and biomedical research [1, 2]. The embryonated chicken egg is favored in embryogenic studies [3, 4] because it allows for easier access and manipulation and is more economical.

Understanding the mechanisms of bone development is highly relevant for poultry farming, where skeletal deformities in long bones can have a substantial economic impact. Insights gained from the chick embryo model also allow for a greater understanding of human bone development and metabolism as well as associated diseases [5–8]. 

Several imaging modalities may be considered for the *in ovo* observation of the live avian embryo: fluorescence microscopy [9], magnetic resonance tomography (MRT) [10], ultrasound [11], and computed tomography (CT) [12]. Both MRT and CT are noninvasive; they do not entail damaging the egg shell. Both imaging modalities provide three-dimensional information at high spatial resolutions, thereby allowing for longitudinal studies and the study of long-term processes (e.g., bone growth and ossification) in the same chick embryo *in ovo*. However, only CT provides sufficient bone contrast.

Unfortunately, the overabundance of generated image data makes the manual analysis of resulting images a time-consuming and tedious task. Furthermore, the visual interpretation of images is error prone and highly subjective. Therefore, automated image analysis systems are highly desirable. The most important tasks of such systems are the automatic detection, segmentation, quantification, and classification of biological structures from various 2D, 3D, and 4D images.

As it mimics human visual perception, the object-oriented image analysis approach based on the Cognition Network Technology (CNT) offers key advantages over pixel-based approaches. Instead of solely relying on pixel information, CNT emulates the segmentation, description, and identification of image objects through context sensitive associations [13]. Based on CNT, rule-based solutions can be created for virtually any question related to image analysis. For rule set creation, a flexible programming language called Cognition Network Language (CNL) has been constructed. Recently, CNT and CNL have been used to solve image analysis tasks in such fields as infection, cell and developmental biology [14–16] and in clinical and preclinical radiology [17, 18].

The extracted image objects and associated properties can be used to train a model for machine learning, which can then be used to automatically classify anatomical units (i.e., bones) in unknown image datasets. As they work on nonlinear problems and can achieve high precision—even with small training sets, support vector machines (SVM) have proven advantageous for object-based image analysis (OBIA) [19].

We demonstrate how automated image analysis and machine learning techniques can be combined to segment microCT images and extract object information for the *in ovo* classification of bones in live chick embryos. 

Using CNT, a rule set that reliably segments *in ovo* microCT images of chick embryos, including those at different stages of incubation, can be created in CNL. The bone objects of interest could be extracted, and their features were used to train an SVM that classifies long bones with high accuracy. To present a potential application of our workflow, we studied long bone growth of chick embryos *in ovo* from day 13 to day 15 of incubation based on daily microCT measurements.

## 2. Methods

### 2.1. Image Data

In the present study, *in ovo* microCT images of chick embryos from day 13 to day 19 of incubation (d13–d19, Hamburger-Hamilton (HH) stages 39–45) were used. The database for machine learning consisted of 27 microCT images (*n* = 4 for d13–d18 and *n* = 3 for d19, Group 1). The database for analyzing long bone growth from d13–d15 consisted of 12 microCT images acquired from the same four eggs. One microCT scan was performed daily over three consecutive days (Group 2).

The microCT images were acquired during a previous study [20] in which the bone metabolism of live chick embryos at different days of incubation was investigated using single and repeated 3D and 4D ^18^F-fluoride microPET. The microCT images were used for the attenuation correction of microPET data.

### 2.2. Embryonated Chicken Eggs

Fertilized *Gallus gallus domesticus* (white leghorn chicken) eggs were obtained from a local breeder (Geflügel GmbH Borna, Germany) and incubated in a forced-air egg incubator (Grumbach BSS300 MP GTFS incubator; Grumbach Brutgeräte GmbH, Germany) at 37.7 ± 0.2°C and a relative humidity of 60 ± 2%. During incubation, eggs were candled and checked daily for viability. Motile embryos were considered healthy. As an additional measure, the Buddy Digital Egg Monitor (Avitronics, UK) was used to confirm a stable heartbeat.

Prior to microPET measurements, a blood vessel of the chorioallantoic membrane was catheterized through a small hole in the shell for injection of the radiotracer ([^18^F]NaF). To ensure the normal development of the experimental chick embryos, the beak length (from where the parasphenoid articulates with the palatine to the tip of the upper bill) was measured on microCT images and compared with controls.

### 2.3. Imaging System and Imaging Protocols

All microCT scans were performed with a Siemens Inveon Small Animal microPET/CT scanner (Siemens Medical Solutions, Siemens Healthcare Molecular Imaging, USA). The final microCT scans were assembled from two consecutive microCT scans (X-ray tube voltage: 80 kV, X-ray tube current: 500 *μ*A) at two animal bed positions. The X-ray detector was operated in a four-by-four pixel binning mode, and 361 projections were acquired per bed position over a 360° rotation of the gantry. Projection slices were sent to a server running the Cobra software (Exxim Computing Corp., USA), where they were reconstructed into images. During reconstruction, the image data were calibrated to Hounsfield Units (HU) and beam hardening correction, as well as a medium noise and ring artifact reduction, was applied. The final microCT images consisted of 604 slices, each 256 × 256 pixels, and isotropic voxel dimensions of 0.216032 mm. For further processing, the image files were converted from a proprietary format into DICOM format using the Siemens Inveon Research Workplace Software (IRW, version 3.0; Siemens Medical Solutions, Siemens Healthcare Molecular Imaging, USA).

### 2.4. Automated Image Segmentation and Feature Extraction

The rule set for automated image segmentation and feature extraction of bone objects was developed with Definiens Developer XD 2 (Definiens AG, Germany) on a computer (Intel Xeon X5650, 2.66 GHz, 24 GB RAM) running Windows XP Professional x64 Edition (Version 2003, Service Pack 2). The rule set is described in detail in the results section.

### 2.5. Data Preparation for Automated Classification

For each egg of Group 1, the image object data extracted during the automated image segmentation step were annotated and classified according to the following categories: humerus, radius, ulna, carpometacarpus, femur, tibiotarsus, and tarsometatarsus. Only clearly discernable bones were classified. All remaining image objects, including those representing clotted or blurred bones or bones that appeared anatomically incorrect (e.g., because of image artifacts), were classified as not of interest (NOI). Finally, all annotated data were combined into a single file that was used to train and test the automated classification process.

For Group 2, the image object data were left unannotated and were further processed as individual files.

### 2.6. Workflow for Long Bone Classification

The automatic long bone classification system was built using the Waikato Environment for Knowledge Analysis (WEKA) machine learning tool [21]. It provides a support vector machine (SVM) implementation based on the sequential minimal optimization (SMO) method [22]. In general, an SVM is a classifier that can separate instances belonging to two classes in a nonlinear space. This is achieved by the kernel trick, which transforms the initial nonlinear problem into a linear one by adjusting the input space. Another key feature of SVMs is that they separate the instances so that a maximal margin between the two classes is achieved. This margin is then expressed by support vectors that define the separating hyperplane. The best parameters, C (the number of support vectors) and *γ* (the variance of the kernel function) for the SVM, were found using a Java implementation of a grid search method [23]. To use such an SVM, it must be trained on a dataset, which results in the specific vectors for the hyperplane. The SVM can then be applied on a test set to classify the long bones. The SVM was trained using the annotated data from the Group 1 eggs. Before training, all input data were standardized. To obtain probability estimates of the classification results, logistic regression models were fitted to the outputs of the SVM.

The accuracy of the trained model was evaluated using 10-fold cross-validation, whereby a set of instances (10 percent of the whole set) are systematically excluded from the training and subsequently used as test set. This is done 10 times with nonoverlapping test sets. To judge the quality of the SVM per bone class, the F-measure gives a good overview, because it combines recall and precision rates [24].

The trained SVM was evaluated based on the classification accuracy for the unlabeled data from Group 2 eggs. For this test, an additional constraint was implemented; each chick embryo could only have two long bones of each kind. Hence, if more than two bones were assigned the same classification, only the two with the highest probability were retained. The superfluous objects were classified as NOI. For assessing classification accuracy, the results were reviewed and the classification error was calculated. For the subsequent analysis of long bone growth, misclassifications were corrected.

### 2.7. The *In Ovo* Analysis of Long Bone Growth of Chick Embryos from d13 to d15

The image object feature *length *was used for the analysis of long bone growth. The feature is derived from the three eigenvalues of a rectangular 3D space with the same volume as the image object and the same proportions of eigenvalues as the image object. The length of an image object is the largest of the eigenvalues.

## 3. Results

### 3.1. Rule Set for Automated Image Segmentation and Feature Extraction

**Figure 1:**
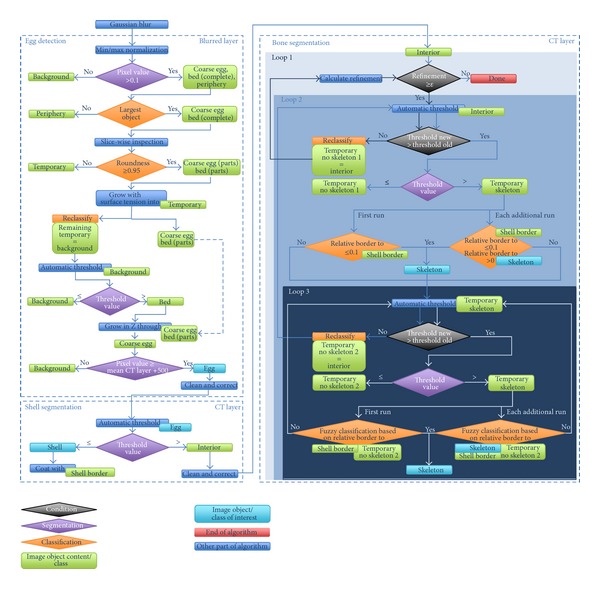
Graphical representation of the complete automated image segmentation procedure. A larger reproduction is provided in the Supplementary Material as Supplemental Figure S1 (see Supplementary material available online at http://dx.doi.org/10.1155/2013/508474).

The image segmentation and feature extraction process is divided into three steps (Figure 1), which are outlined next: egg detection, shell segmentation, and bone segmentation. 

#### 3.1.1. Egg Detection

**Figure 2:**
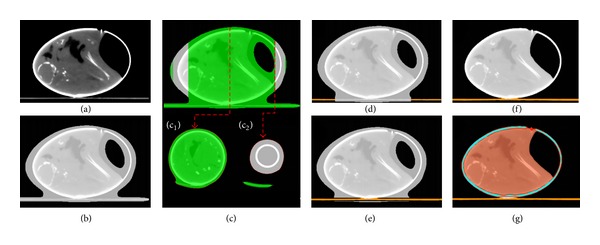
Steps of *Egg Detection* and *Shell Segmentation*. (a) input image; (b) initial segmentation after coarse Gaussian blur; (c) slicewise inspection and search for round image objects; (c_1_) example of an image object (green) below the defined threshold for roundness; (c_2_) example of image objects above (white) and below (green) the defined threshold for roundness; (d) parts of the animal bed (orange) were removed by retaining only the round (white) image objects from the previous step and reexpanding them by applying a stringent surface tension criterion to prevent expansion too far back into the animal be; (e) the animal bed was separated from the background and expanded along the *z*-axis through the *Coarse Egg/Bed (Parts)* (white) object; (f) the *Coarse Egg* (white) object was segmented using a fixed threshold and further smoothed by three consecutive expansion and reduction operations; (g) using an automatically calculated threshold, the refined *Egg* object was segmented into *Shell* (light blue) and *Interior *(light red). The *Shell* object was surrounded by two additional layers of pixels (*Shell Border, red*).

Each input microCT image comprised the egg, the animal bed, and the background (i.e., air) (Figure 2(a)). Images occasionally contained anesthetic equipment (i.e., tubing or nozzles), which had to be removed. The first step of image processing involves separating the background from the rest of the image content. Therefore, a large Gaussian blur (kernel size: 51 × 51 × 99) was applied. The resulting layer was min-max normalized, and every pixel with a value less than or equal to 0.1 was discarded as background (Figure 2(b)). All other pixels were kept as image objects. 

To exclude additional periphery, only the largest image object (i.e., the egg and the complete animal bed object) was retained. Next, this *Coarse Egg (Complete)/Bed (Complete)* object needed to be segmented into its two components. Since the carbon fiber bed and the interior of the egg have similar pixel values, this separation cannot be performed based solely on these values. Therefore, a modeling approach that exploits morphological differences between the animal bed and the egg was developed. This approach is based on the knowledge that the egg is axially aligned in the field of view (FOV) of the microCT scanner and that in an axial view an egg is much rounder than the animal bed. 

To separate the egg from the bed, the *Coarse Egg/Bed (Complete)* object was first split into a series of 2D slices (Figure 2(c)). On each slice, all parts of this object below a certain degree of roundness (calculated as the quotient of the radius of the largest enclosed ellipse divided by the radius of the smallest enclosing ellipse) were reclassified as *Temporary* objects (Figure 2 c_1_). Using a pixel-based grow operation, the remaining round objects (Figure 2 c_2_) were first expanded along the *z-*axis (i.e., from the blunt to the pointed end of the egg) and then along the *x-* and *y*-axes into the *Temporary* objects. 

During the growth process, a stringent surface tension criterion was applied to prevent the new object from growing back into the initial *Coarse Egg/Bed (Complete)* object. The result of this step was the *Coarse Egg/Bed (Parts)* object (Figure 2(d)). The remaining *Temporary* objects were reclassified as background. Consequently, the background consisted only of air and the animal bed, a fact that could then be used to help remove the remaining part of the bed from the *Coarse Egg/Bed (Parts)* object. The strategy was to model the animal bed by segmenting the background into bed and air and then expand the bed through the *Coarse Egg/Bed (Parts) *object using the grow operation. 

To separate the two image parts of the background, a threshold was calculated by using the *automatic threshold *function (AT) on the original unfiltered CT layer. Based on a combination of histogram and homogeneity measurements, this function calculates a pixel value such that intensity differences and heterogeneity increase to a maximum between the resulting pixel subsets. In this case, the respective subsets were the animal bed and air. The background was segmented using the calculated threshold. All pixels with values above the threshold were then classified as *Bed*. This image object was then expanded along the *z*-axis through the *Coarse Egg/Bed (Parts) *object. As a result, all affected pixels, and thus the animal bed, were reclassified as *Background* and removed from this object (Figure 2(e)) resulting in the *Coarse Egg *object.

The last step of the egg detection step was to refine the remaining *Coarse Egg* object into its final shape. The object consisting of air and the actual egg was separated into these two components by applying another segmentation process that used a fixed intensity threshold (mean pixel value of the egg object + 500) on the unfiltered image layer. The resulting object was further smoothed into the final egg object using three expansion and reduction steps (i.e., grow and shrink operations) (Figure 2(f)).

#### 3.1.2. Shell Segmentation

The aim of this step was to separate the eggshell from the egg interior. Here, the AT function could be reapplied, because the shell and the interior form two well-separated pixel subsets. The subsequent segmentation using the calculated threshold value resulted in a *Shell* object and an object representing the *Interior* of the egg (Figure 2(g)). Additionally, the *Shell *object was surrounded by two layers of pixels classified as *Shell Border.*

#### 3.1.3. Bone Segmentation

In this step, the skeleton was separated from the rest of the egg interior. Because calcified bones have considerably high pixel values that form a pixel subset distinct from the egg interior, the AT function could also be applied extensively in this step. The AT function extends enough robustness to the entire rule set so that bones and bony structures are correctly segmented for eggs from d13 to d19. However, some additional measures needed to be taken for the correct segmentation of bones located close to the shell. Here, the AT function fails to directly calculate the best separating threshold, and consequently, initial segmentation often leads to large image objects that are attached to the shell and which need to be further treated.

A possible solution that provides robustness, as well as a high segmentation quality, was implemented in three nested loops that perform repeated automatic threshold calculation, image segmentation, and segmentation refinements (Figure 1). 

The outer loop was used for global refinement and to control if the segmentation steps performed in the two inner loops contributed a substantial amount of new pixels to new or existing *Skeleton* image objects. The complete bone segmentation step was terminated when the last round of segmentation and classification performed by the two inner loops did not increase the number of pixels classified as *Skeleton* by 0.005% or greater.

In the first inner loop, a threshold was calculated, and the *Interior* was segmented into *Temporary Skeleton* and *Temporary No Skeleton 1* using that threshold. Following segmentation, only resulting *Temporary Skeleton* image objects were further processed. If it was the first run of the outer loop, all *Temporary Skeleton* image objects with a relative border to *Shell Border* smaller than or equal to 0.1 were classified as *Skeleton*. In all subsequent runs of the outer loop, a second condition was introduced; new *Temporary Skeleton* image objects also needed to share a relative border with existing *Skeleton* objects in order to also be classified as *Skeleton*.

All remaining *Temporary Skeleton* that could not be classified as *Skeleton* because they did not satisfy the border conditions were then fed into the second inner loop for the refinement of segmentation and the extraction of additional *Skeleton* image objects. The second inner loop had the same basic functional principle as the first inner loop. Using an automatically calculated threshold, the *Temporary Skeleton* image objects were further segmented into *Temporary Skeleton* and *Temporary No Skeleton 2* objects. However, unlike the first inner loop, the classification of the *Temporary Skeleton* objects was now performed using the relative border to the *Shell Border* and to *Temporary No Skeleton 2 *as measurements combined in a fuzzy set with a linear or sigmoidal membership function. The second inner loop was exited when the AT function could not calculate a new threshold (i.e., the best separating value was reached). All remaining *Temporary No Skeleton 2 *objects were then reclassified as *Interior* and fed back into the first inner loop.

**Figure 3:**
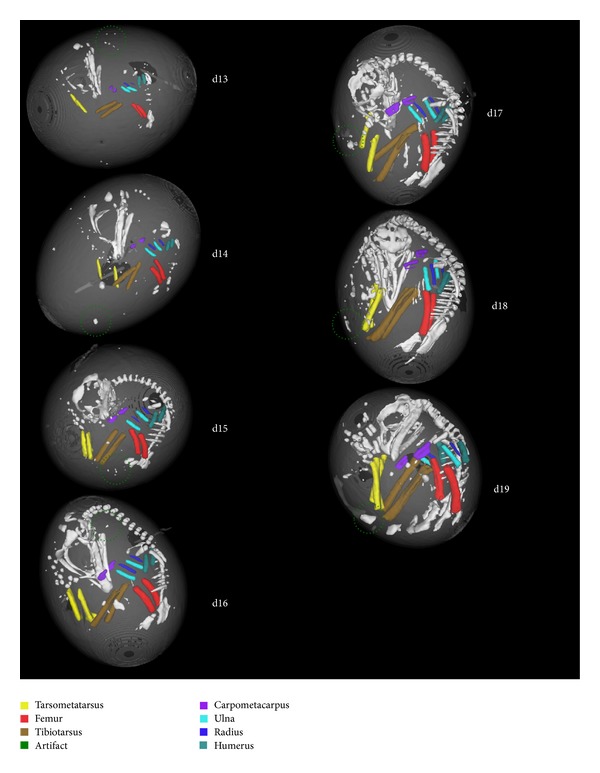
Segmentation and classification of microCT images of Group 1 chick embryos from d13 to d19. The bone names and their corresponding colors are presented. Image artifacts are circled in green.

The first inner loop was exited when a better separating threshold could not be calculated. The number of pixels added to existing *Skeleton* image objects or representing new *Skeleton* image objects was then calculated in order to confirm if the termination condition of the outer loop was satisfied. If the refinement was above the threshold, the *Temporary No Skeleton 2 *objects were combined, reclassified as *Interior,* and subjected to another round of bone segmentation. Otherwise, after satisfying the termination condition of the outer loop, the final result of the bone segmentation step was a set of *Skeleton* image objects representing the chick embryo skeleton and a number of image artifacts (Figure 3). For each of these image objects, the following feature values were calculated and exported into a CSV file: day of incubation, asymmetry, border length, compactness, elliptical fit, length, thickness, width, length/thickness, length/width, volume in relation to the total volume of all extracted bone objects, radius of largest enclosed ellipse, radius of smallest enclosing ellipse, rectangular fit, roundness, shape index, mean of the CT image layer, standard deviation of the CT image layer, skewness of the CT image layer, minimal pixel value of the CT image layer, maximal pixel value of the CT image layer, number of other bone objects within a range of 50 pixels, distance to the nearest *Skeleton* object, and distance to *Shell*.

The typical run time of the rule set was between 6 and 15 min depending on the number of bones, bony objects, and artifacts in the image. 

### 3.2. Training the SVM for Long Bone Classification

A total of 2951 annotated object instances extracted from the images of Group 1 were used for training the SVM. The grid search yielded an optimal setting of 1.25 for the C and 0.1125 for *γ*. After 10-fold cross-validation, the model proved to be highly accurate (Table 1) and was able to correctly classify 98.6% of the instances. The femur can be most accurately identified (F-measure 0.981), while the correct annotation of bones of the carpometacarpus is slightly more challenging (F-measure 0.882). The largest class (NOI) can also be readily separated from the other bone types. This is integral to the automated annotation of datasets not having bone name labels (unlabeled dataset). The highest numbers of false positives (15) and false negatives (16) were, however, identified during classification of the NOI objects (Table 2).

**Figure 4:**
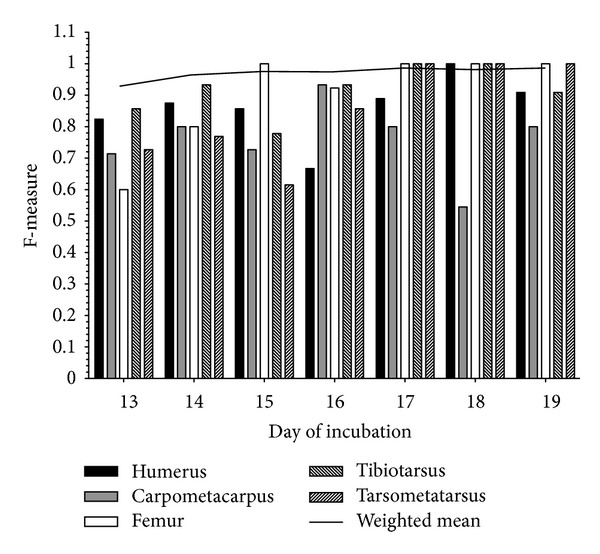
Bar chart of the classification accuracy of SVMs trained for single days of incubation. To evaluate the classification performance for a single day of incubation, an SVM was trained on all objects but the image objects belonging to the corresponding day. The resulting SVM was then applied to these excluded objects. The overall classification accuracy increases with the day of incubation.

To evaluate the classification performance for a single day of incubation, an SVM that leaves out bones for that specific day, was trained on all remaining objects. The resulting SVM was then applied to the image objects of the excluded day of incubation. In general, the classification accuracy increases with the day of incubation (Figure 4). Bones become more calcified the longer the egg is incubated, thus allowing for improved differentiation. However, the classification accuracy is dependent on bone type. While the femur can easily be identified, annotation of the carpometacarpus can be problematic. Nevertheless, the intrinsic information from the other days facilitates accurate annotation. Hence, this model can also be used to transfer knowledge between different days of incubation.

The performance of the trained SVM model was evaluated on an unlabeled test set. The SVM correctly classified 98.6% of the 1203 object instances that were extracted from the microCT images of Group 2 eggs. The best classification reliability (F-measure 0.979) was again obtained for the femur (Table 3). In contrast to Group 1, the ulna (F-measure 0.884), not the carpometacarpus (F-measure 0.933), was the bone with the lowest classification reliability. Interestingly, for the femur, the *Precision* is higher for the test set than for the training set (i.e., the application of the model to unknown datasets is highly confident). As with Group 1, the NOI objects from Group 2 also yielded the highest number of false positives (i.e., 12). A maximum of three false negatives were identified during classification of the humerus, radius, carpometacarpus, and tibiotarsus (Table 4).

### 3.3. *In Ovo* Measurement of Long Bone Growth of Chick Embryos from d13 to d15

**Figure 5:**
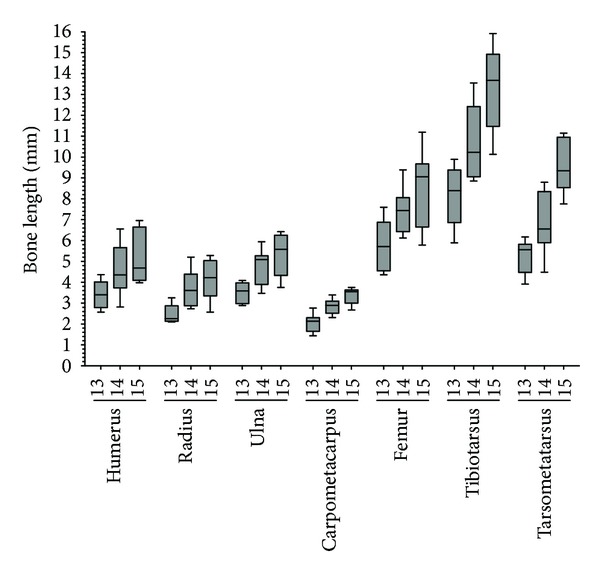
Box and whisker plot of long bone lengths of chick embryos from Group 2. The numerical values are presented in Supplemental Table S1.

Crural bones are much longer (Figure 5) and have a much higher rate of growth than aral bones (Table 5). The highest increase in length (2.902 mm/day) was recorded for the tarsometatarsus from d14 to d15, while for the same interval, the radius had the smallest increase (0.504 mm/day).

## 4. Discussion

Noninvasive microCT offers quantitative imaging with high spatial resolution as well as the possibility of repeatedly imaging and measuring the same chick embryo *in ovo. *The excellent bone contrast can be used to investigate bone-related questions, for example, bone formation (in conjunction with automated image segmentation methods [25]) and tumor-induced bone destruction [26]. To our knowledge, these techniques had not yet been applied to the *in ovo* quantification of bone growth of live chick embryos. We developed an approach for the automated segmentation of *in ovo* microCT images from live chick embryos using OBIA, followed by automated classification of the extracted image objects. As automated routines heavily reduce processing time, more images can be analyzed within the same timespan. Moreover, such systems minimize observer bias. The Definiens Developer XD rule set for automated image segmentation was developed using only one egg at d18. However, it proved to be robust enough to successfully segment the skeleton from the rest of the egg and its periphery on microCT images for eggs from d13 to d19 without having to adjust parameters for incubation day. Thus, the approach could effectively manage variations in bone size and calcification. In addition for determining feature values for single bones, the segmentation and classification results could be also used to provide an excellent 3D *in ovo* visualization of the developing chick embryo (Figure 3).

The high classification accuracy using an SVM greatly facilitates the classification of objects extracted from microCT images, although bones with a low classification probability should be reviewed to avoid corruption of length measurements. An iterative approach comprising repeated classification and feature value calculation could, however, refine the existing classification and enable the classification of previously unclassified objects. In the first step, only objects with high classification probabilities would be classified. Based on this initial classification, new feature values (e.g., distances) could be calculated for unclassified objects in a subsequent step. In turn, these values could be used to train a new and extended classifier.

Long bone growth during chick embryogenesis has been extensively studied under normal conditions [27–29] as well as under various environmental influences such as insecticides [30], increased temperature [31], and acceleration [32]. However, none of these studies provided *in vivo* data; the chick embryos were sacrificed, removed from the egg, and fixed. Bone lengths were either measured while bones were still attached to the limb or after they were dissected from adherent tissue.

Therefore, the long bone length measurements presented in this study represent the first true *in vivo *data. While our results deviate from those of the aforementioned *ex ovo* and *ex vivo* measurements, there were also discrepancies among those studies. For example, for d15 we measured a mean femur length of 8.574 mm, Alfonso-Torres et al. [28] reported a length of 14.51 mm (by polynomial regression), and Hammond et. al reported a length of 11.25 mm (Figure 4(b) in [31]). In addition to human bias, these apparent differences may arise from variations in breeder age [28] and incubation temperature [31] as well as bone shrinkage resulting from fixation or preparation [33]. Our methodology imposes additional constraints that should be considered when comparing our measured lengths to those of classical studies. As CT has poor cartilage contrast, only areas of sufficiently mineralized bone can be imaged and measured. The image analysis software also calculates lengths differently than direct measurement (i.e., via a ruler or calipers).

We present an innovative approach for the automated segmentation, classification, quantification, and visualization of microCT images. MicroCT offers the possibility to perform longitudinal studies and thereby provides unique insights into the morpho- and embryogenesis of the live chick embryo. By using OBIA, image parts (e.g., bones) may be extracted from an image in order to calculate various morphometric feature values. These can subsequently be used to train a classifier that can identify image objects based on these unique values. Despite a high classification accuracy, some misclassifications must still be manually corrected in order to yield statistically valid results. Human expertise is therefore still required for the interpretation and validation of results. Nevertheless, automated systems can greatly expedite image analysis and reduce observer bias.

## Supplementary Material

Supplemental Figure 1 is a larger reproduction of Figure 1 and shows a graphical representation of the complete automated image segmentation procedure.
Supplemental Table 1 shows the numerical values that are the basis of the box and whisker plot presented Figure 5.

## Figures and Tables

**Table 1:** Classification accuracy of the SVM after 10-fold cross-validation on the training data from Group 1 eggs.

Classes	True positive rate	False positive rate	Precision	Recall	F-measure	ROC-area
Humerus	0.941	0.003	0.842	0.941	0.889	0.999
Radius	0.929	0.001	0.929	0.929	0.929	0.996
Ulna	0.918	0.001	0.938	0.918	0.928	0.979
Carpometacarpus	0.865	0.002	0.900	0.865	0.882	0.988
Femur	1.000	0.001	0.962	1.000	0.981	1.000
Tibiotarsus	0.925	0.000	0.980	0.925	0.951	0.999
Tarsometatarsus	0.941	0.001	0.960	0.941	0.950	0.991
Not of interest (NOI)	0.994	0.043	0.994	0.994	0.994	0.996
**Weighted avg.**	**0.986**	**0.038**	**0.987**	**0.986**	**0.986**	**0.996**

**Table 2:** Confusion matrix of the SVM after 10-fold cross-validation on the training data from Group 1 eggs.

Classes	Classified as							
	a	b	c	d	e	f	g	h
a = humerus	48	0	0	0	0	0	0	3
b = radius	0	39	1	0	0	0	0	2
c = ulna	1	0	45	0	0	0	0	3
d = carpometacarpus	1	0	1	45	0	0	0	5
e = femur	0	0	0	0	51	0	0	0
f = tibiotarsus	0	0	0	0	1	49	2	1
g = tarsometatarsus	0	0	0	0	1	1	48	1
h = not of interest (NOI)	7	3	1	5	0	0	0	2586

**Table 3:** Classification accuracy of the SVM on the test data from Group 2 eggs.

Classes	True positive rate	False positive rate	Precision	Recall	F-measure	ROC-area
Humerus	0.875	0.000	1.000	0.875	0.933	0.966
Radius	0.938	0.002	0.882	0.938	0.909	0.999
Ulna	0.864	0.002	0.905	0.864	0.884	0.999
Carpometacarpus	0.875	0.000	1.000	0.875	0.933	0.965
Femur	0.958	0.000	1.000	0.958	0.979	0.975
Tibiotarsus	0.875	0.000	1.000	0.875	0.933	0.978
Tarsometatarsus	0.905	0.001	0.950	0.905	0.927	0.980
Not of interest (NOI)	0.999	0.077	0.989	0.999	0.994	0.989
**Weighted avg.**	**0.986**	**0.068**	**0.986**	**0.986**	**0.988**	**0.989**

**Table 4:** Confusion matrix of the SVM on the test data from the Group 2 eggs.

Classes	Classified as							
	a	b	c	d	e	f	g	h
a = humerus	21	0	0	0	0	0	0	3
b = radius	0	15	0	0	0	0	0	1
c = ulna	0	2	19	0	0	0	0	1
d = carpometacarpus	0	0	1	21	0	0	0	2
e = femur	0	0	0	0	23	0	0	1
f = tibiotarsus	0	0	0	0	0	21	1	2
g = tarsometatarsus	0	0	0	0	0	0	19	2
h = not of interest (NOI)	0	0	1	0	0	0	0	1047

**Table 5:** Bone growth rates of the Group 2 chick embryos.

Classes	d13 to d14 (mm/day)	d14 to d15 (mm/day)
Humerus	1.186	0.626
Radius	1.245	0.504
Ulna	1.323	0.534
Carpometacarpus	0.802	0.529
Femur	1.625	1.174
Tibiotarsus	2.491	2.688
Tarsometatarsus	1.473	2.902
