# Supplementary material for: Automated Segmentation and Object Classification of CT Images: Application to In Vivo Molecular Imaging of Avian Embryos
Source: Int J Biomed Imaging. 2013 Aug 12;2013:508474. doi: 10.1155/2013/508474 (PMC3753761; doi:10.1155/2013/508474)
Supplement: Supplementary file 1 — Supplemental Figure 1 is a larger reproduction of Figure 1 and shows a graphical representation of the complete automated image segmentation procedure. Supplemental Table 1 shows the numerical values that are the basis of the box and whisker plot presented Figure 5. [file 508474.f1.pdf]

International Journal of Biomedical Imaging

Automated Segmentation and Object Classification of CT Images: Application to *In Vivo* Molecular Imaging of Avian Embryos

Supplementary Material

Alexander Heidrich,[1] Jana Schmidt,[2] Johannes Zimmermann,[3] Hans Peter Saluz[1,4]

[1] *Department of Cell and Molecular Biology, Leibniz Institute for Natural Product Research and Infection Biology – Hans Knöll Institute, Jena, Germany*
[2] *Institut für Informatik / I12, Technische Universität München, Garching b. München, Germany*
[3] *Definiens AG, München, Germany*
[4] *Friedrich Schiller University of Jena, Jena, Germany*
Correspondence to: Hans Peter Saluz; *e-mail:* hanspeter.saluz@hki-jena.de

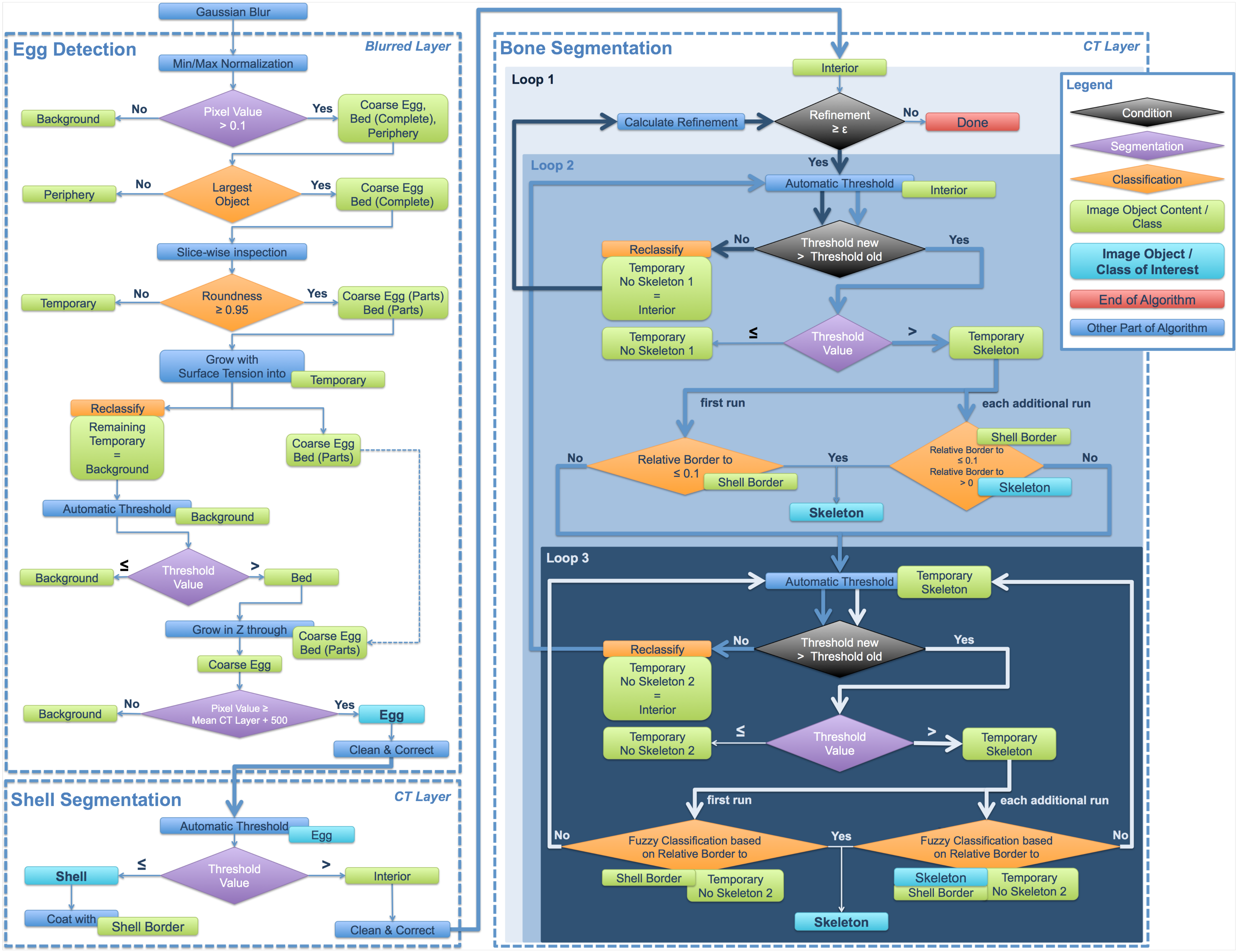

Supplemental Fig. S1 Graphical representation of the complete automated image segmentation procedure

Supplemental Table S1. Bone lengths of Group 2 chick embryos

Classes	d13		d14		d15	
	**Mean (mm ± SD)**	**n**	**Mean (mm ± SD)**	**n**	**Mean (mm ± SD)**	**n**
Humerus	3.425 ± 0.678	8	4.612 ± 1.208	8	5.238 ± 1.276	8
Radius	2.454 ± 0.472	5	3.699 ± 0.937	6	4.202 ± 1.023	5
Ulna	3.497 ± 0.475	8	4.820 ± 0.853	7	5.354 ± 0.981	7
Carpometacarpus	2.053 ± 0.427	8	2.856 ± 0.363	8	3.385 ± 0.398	8
Femur	5.774 ± 1.232	8	7.400 ± 1.063	8	8.574 ± 1.830	8
Tibiotarsus	8.200 ± 1.408	8	10.691 ± 1.801	8	13.380 ± 1.984	8
Tarsometatarsus	5.286 ± 0.804	8	6.758 ± 1.486	8	9.660 ± 1.349	5
